# Increased Carbon Dioxide Concentration Improves the Antioxidative Properties of the Malaysian Herb Kacip Fatimah (*Labisia pumila* Blume)

**DOI:** 10.3390/molecules16076068

**Published:** 2011-07-20

**Authors:** Mohd Hafiz Ibrahim, Z.E. Jaafar Hawa

**Affiliations:** Department of Crop Science, Faculty of Agriculture, University Putra Malaysia, Serdang 43400, Selangor, Malaysia; Email: mhafizphd@gmail.com (M.H.I.)

**Keywords:** elevated CO_2_, secondary metabolites, soluble carbohydrate, gluthatione, antioxidative properties

## Abstract

A randomized complete randomized design (RCBD) 3 by 3 experiment was designed to investigate and distinguish the relationships among production of secondary metabolites (total phenolics, TP; total flavonoids, TF), gluthatione (GSH), oxidized gluthatione (GSSG), soluble carbohydrate and antioxidant activities of the Malaysian medicinal herb *Labisia pumila* Blume under three levels of CO_2_ enrichment (400, 800 and 1,200 µmol mol^−1^) for 15 weeks. It was found that the treatment effects were solely contributed by interaction of CO_2_ levels and secondary metabolites distribution in plant parts, GSH, GSHH and antioxidant activities (peroxyl radicals (ROO), superoxide radicals (O_2_), hydrogen peroxide (H_2_O_2_) and hydroxyl radicals (OH). The records of secondary metabolites, glutahione, oxidized gluthathione and antioxidant activities in a descending manner came from the leaf enriched with 1,200 µmol/mol CO_2_ > leaf 800 µmol/mol CO_2_ > leaf 400 µmol/mol CO_2_ > stem 1,200 µmol/mol CO_2_ > stem 800 µmol/mol CO_2_ > stem 400 µmol/mol CO_2_ > root 1,200 µmol/mol CO_2_ > root 800 µmol/mol CO_2_ > root 400 µmol/mol CO_2_. Correlation analyses revealed strong significant positive coefficients of antioxidant activities with total phenolics, flavonoids, GSH and GSHH indicating that an increase in antioxidative activity of *L. pumila* under elevated CO_2_ might be up-regulated by the increase in production of total phenolics, total flavonoids, GSH, GSHH and soluble sugar. This study implied that the medicinal potential of herbal plant such as *L. pumila* can be enhanced under elevated CO_2_, which had simultaneously improved the antioxidative activity that indicated by the high oxygen radical absorbance activity against ROO, O_2_, H_2_O_2_, and OH radicals.

## 1. Introduction

Carbon dioxide is one of the most limiting factors in photosynthesis. The possibility of improving photosynthesis in crops through CO_2_ enrichment has interested agriculturists for many years [1]. CO_2_ enrichment has been shown to increase plant growth, development, and yield of agricultural crops, and this response is a function of CO_2_ concentration and duration [2,3]. Elevated CO_2_ concentrations enhanced vegetative growth, biomass increment, carbohydrate accumulation, fruit productivity, and quality in plants under glass-house conditions [4]. Crops under enriched CO_2_ atmosphere acquire positive features with enhanced plant adaptation and growth. The greatest advantages of CO_2_ enrichment is in the enhancement of photosynthetic capacity, particularly under adverse climatic conditions and this would become most apparent in the vegetative growth of young plants [5,6].

The antioxidant properties in food have been a focus of interest in recent years due to the health maintenance functions of these components that can help reduce the risk of chronic diseases such as cancer, hypertension and diabetes. This is attributed to the high scavenging activity of antioxidants towards free radicals that are usually associated with these diseases [7,8]. It is currently known that phenolic acids and flavonoids are antioxidants with high anti-inflammatory and anticarcinogenic activities [7,9,10]. It is also known that phenolics and flavonoids can function as free radical scavengers, reducing agents and quenchers of singlet oxygen formation [11]. The components of polyphenols have been proven to have important roles in the regulation of cancer and disease development in the human body [12,13]. For instance, in green tea the effectiveness of the plant extract to inhibit cancer and tumor invasion is due to the high phenolics and flavonoids content in the plant [14].

Rising levels of atmospheric CO_2_ can alter plant growth and the partitioning of secondary metabolites [8]. An increase in C-based secondary metabolites frequently occurs when environmental conditions promote the accumulation of non-structural carbohydrates (TNC) in plants. Elevated atmospheric CO_2_ concentrations often increase TNC concentrations in plants and thus possibly stimulate secondary metabolism [15]. Idso *et al.* [16] evaluated the response of the tropical spider lily (*Hymenocallis littoralis*) to elevated levels of atmospheric CO_2_ over four growing seasons and found that a 75% increase in the air’s CO_2_ concentration produced an 8% increase in pancratistatin, an 8% increase in *trans*-dihydronarciclasine, and a 28% increase in narciclasine that are effective against lymphocytic leukemia and ovary sarcoma [17]. In the early studies of Barbale [18] and Madsen [19,20], a tripling of the atmospheric CO_2_ concentration produced a modest (7%) increase in antioxidant activity in the leaves and fruits of tomato plants. Tajiri [21] and Schwanz *et al.* [22] found ascorbate concentrations in the sun leaves of sour orange trees to be signiﬁcantly enhanced by a 600 µmol/mol CO_2_ increase in the air. It can be concluded that exposure of plant to high levels of CO_2_ can increase the production of secondary metabolites and antioxidant activity.

Rao *et al.* [23] observed that atmospheric CO_2_ enrichment increases NADPH content and leads to the maintenance of higher activities of antioxidant enzymes such as glutathione reductase. Since this enzyme is primarily responsible for the high redox states of both glutathione and ascorbate [24], this phenomenon may well be operative among a host of antioxidants, and especially among vitamins. It is interesting to note, however, that several studies that have looked at the effects of atmospheric CO_2_ enrichment on plant antioxidative compounds have found increased concentrations of these plant constituents as well as increase in activity against peroxyl radicals (ROO), superoxide radicals (O_2_), hydrogen peroxide (H_2_O_2_) and hydroxyl radicals (OH) [25]. Exposing medicinal plants to CO_2_ may give positive response to increased antioxidative properties. Contrastingly, several researchers have found reduction in antioxidative activity under elevated CO_2_ exposure [26,27,28,29] probably due to differences in plant species responses and other interacting microenvironment.

*Labisia pumila* is good source of natural antioxidants [30]. In addition, the plant is also rich in anthocyanins, flavonoids, and phenolic acids [31,32]. Results of elevated CO_2_ exposure on total phenolics and flavonoids have been reported by Ibrahim *et al.* [33], however, there is no available information on the effect of CO_2_ concentration on the production of secondary metabolites and scavenging capacity against active oxygen species for this plant species. Hence, this study was performed to evaluate the effects of elevated carbon dioxide enrichment on the concentration of phenolics, flavonoids, glutathione (GSH), gluthatione oxidase (GSSG) and antioxidative activity in the ethanolic extracts of *L. pumila*. The relationships among parameters of total phenolics, flavonoids, gluthatione, glutatione oxidase and antioxidative activity [peroxyl radicals (ROO), superoxide radicals (O_2_), hydrogen peroxide (H_2_O_2_) and hydroxyl radicals (OH·)] were also established.

## 2. Results and Discussion

### 2.1. Total Phenolics and Flavonoids Profiling

Accumulation of total phenolics and flavonoids in *L. pumila* was influenced by the interaction effect between CO_2_ and plant parts (P ≤ 0.01; Table 1). Generally, total phenolics was observed to be higher in the leaf at 1,200 µmol/mol CO_2_ (1.259 mg gallic acid/g dry weight) followed by leaf-800 µmol/mol CO_2_ (1.167 mg gallic acid/g dry weight), leaf-400 µmol/mol CO_2_ (0.835 mg gallic acid/g dry weight), stem-1,200 µmol/mol CO_2_ (0.862 mg gallic acid/g dry weight), stem-800 µmol/mol CO_2_ (0.678 mg gallic acid/g dry weight), stem-400 µmol/mol CO_2_ (0.531 mg gallic acid/g dry weight), root-1,200 µmol/mol CO_2_ (0.554 mg gallic acid/g dry weight), root-800 µmol/mol CO_2_ (0.343 mg gallic acid/g dry weight) and root-400 µmol/mol CO_2_ (0.311 mg gallic acid/g dry weight). Total flavonoids content followed the same trend with total phenolics where the highest total flavonoids was observed in leaf at 1,200 µmol/mol CO_2_ that registered 0.276 mg rutin/g dry weight and the lowest was in the root at 400 µmol/mol CO_2_ that contained only 0.052 mg rutin/g dry weight. The present results are in agreement with those from Norhaiza *et al.* [30] and Karimi *et al.* [34] where they found the highest bioactive compound (total phenolics and flavonoid) of *L. pumila* was highest accumulated in the leaf than other plant parts. Furthermore, from previous study by Ibrahim *et al.* [33] it was shown that the enrichment of *L. pumila* to elevated CO_2_ was able to enhance the production of total phenolics and flavonoids content, especially in the leaves compared to other plant parts. In this study it was shown that the increased production of secondary metabolites was due to an increase in the production of total non- structural carbohydrate (TNC) that up-regulated the production of secondary metabolites. The enhanced production of plant secondary metabolites under high levels of CO_2_ have also been observed in *Zingiber officianale*, *Betula pendula* and *Fragaria annassa* [25,35,36]. The present result indicated that enrichment of *L. pumila* with high CO_2_ can up-regulate the production of secondary metabolites. The high total phenolics and flavonoids content in the plant has been shown to have anticancer properties and also have an application to use as antibiotics, antidiarrhea, antiulcer and antiinflammatory agents, as well as in the treatment of diseases such as hypertension, vascular fragility, allergies and hyperchlolestrolemia [37,39].

**Table 1:** Total phenolics and flavonoids contents in different parts of *L. pumila *under different CO_2_ concentration.

CO_2_ levels (µmol/mol)	Plant parts	Total phenolics (mg/g gallic acid dry weight)	Total flavonoid (mg/g rutin dry weight)
**400**	Leaf	0.835 ± 0.017b	0.111 ± 0.018c
	Stem	0.531 ± 0.022d	0.071 ± 0.022d
	Root	0.311 ± 0.018e	0.052 ± 0.032d
**800**	Leaf	1.167 ± 0.023a	0.247 ± 0.017a
	Stem	0.678 ± 0.021c	0.143 ± 0.023b
	Root	0.343 ± 0.011c	0.067 ± 0.024d
**1200**	Leaf	1.259 ± 0.032a	0.276 ± 0.021a
	Stem	0.862 ± 0.027b	0.165 ± 0.032b
	Root	0.554 ± 0.041d	0.085 ± 0.031d

All analyses are mean ± standard error of mean (SEM), N = 18. Means not sharing a common single letter were significantly different at P ≤ 0.05.

### 2.2. Antioxidant Activity against Peroxyl Radicals (ROO), Superoxide Radicals (O_2_), Hydrogen Peroxide (H_2_O_2_) and Hydroxyl Radicals (OH)

The antioxidant activity of ROO, O_2_, H_2_O_2_ and OH was influenced by the interaction effects between CO_2_ and plant parts (P ≤ 0.01; Table 2). In ROO the highest antioxidant activity was recorded in the leaf at 1,200 µmol/mol CO_2_ (145.67 µmol TE/g dry weight) followed by leaf-800 µmol/mol CO_2_ (142.32 µmol TE/g dry weight), leaf-400 µmol/mol CO_2_ (132.61 µmol TE/g dry weight), stem-1,200 µmol/mol CO_2_ (121.21 µmol TE/g dry weight), stem-800 µmol/mol CO_2_ (142.32 µmol TE/g dry weight), stem-400 µmol/mol CO_2_ (100.31 µmol TE/g dry weight), and in the root at 400 µmol/mol CO_2_ (94.32 µmol TE/g dry weight). For O_2_, H_2_O_2_ and OH, the antioxidant activity showed similar pattern as ROO. In OH antioxidant, the highest activity was recorded in the leaf at 1,200 µmol/mol CO_2_ (66.54 µmol chlorogenic acid/g dry weight) and the lowest was in root at 400 mol/mol CO_2_ that only recorded 18.65 µmol chlorogenic acid/g dry weight. In this study, elevation of CO_2_ over 800 to 1,200 µmol/mol CO_2_ all resulted in an increase in values of oxygen radical absorbance capacity. The highest CO_2_ enrichment (1,200 µmol/mol CO_2_) yielded *L. pumila* plants with the most ROO, as well as O_2_, H_2_O_2_, OH absorbance capacity. This data indicated that *L. pumila* grown with CO_2_ enrichment had high scavenging activity for chemically generated active oxygen species [25]. Correlation analyses in Table 3 show that the increase in antioxidative properties might be up-regulated by the increase in total phenolics and flavonoids content of plant under elevated CO_2_. All the antioxidant properties were observed to have strong significant positive correlations with total phenolics and flavonoids. This imply that the increase in total phenolics and flavonoids under elevated CO_2_ might be associated with increased antioxidant capacities that may allow quenching of the excited state of active oxygen species [39,40].

**Table 2:** Antioxidant activity against Peroxyl Radicals (ROO), Superoxide Radicals (O_2_), Hydrogen Peroxide (H_2_O_2_) and Hydoxyl Radicals in different part of *L. pumila* under different CO_2_ concentration.

CO_2_ levels (µmol mol^−1^)	Plants Parts	ROO (µmol TE/g dry wt) ^a^	O_2_ (µmol α-tocopherol/g dry wt) ^b^	H_2_O_2_ (µmol ascorbate/g dry wt) ^c^	OH (µmol chlorogenic acid/g dry wt) ^d^
	Leaf	132.61 ± 6.4a	39.22 ± 0.7b	19.45 ± 0.5a	43.22 ± 2.7b
**400**	Stem	100.31 ± 5.3c	27.43 ± 0.5c	12.32 ± 0.1c	33.21 ± 3.2c
	Root	94.32 ± 4.2d	18.91 ± 1.6e	9.23 ± 0.3e	18.65 ± 1.9d
	Leaf	142.32 ± 3.4a	44.56 ± 2.3a	23.34 ± 0.2a	56.73 ± 4.2a
**800**	Stem	112.32 ± 2.3c	32.12 ± 1.7c	15.43 ± 0.1b	40.12 ± 5.6b
	Root	87.34 ± 1.7d	21.32 ± 2.7d	12.34 ± 0.6c	19.34 ± 6.2d
	Leaf	145.67 ± 2.3a	56.34 ± 3.6a	26.54 ± 0.4a	66.54 ± 4.2a
**1200**	Stem	121.21 ± 7.8b	39.23 ± 5.6b	17.54 ± 1.3b	53.21 ± 1.6a
	Root	89.34 ± 2.7d	24.56 ± 7.3d	13.32 ± 0.4c	21.34 ± 0.8d

All analyses are mean ± standard error of mean (SEM), N = 18. Means not sharing a common single letter were significantly different at P ≤ 0.05. ^a^ Data expressed as micromoles of Trolox equivalent per gram dry weight; ^b^ Data expressed as micromoles of α-tocopherol equivalent per gram dry weight; ^c^ Data expressed as micromoles of ascorbate equivalent per gram dry weight; ^d^ Data expressed as micromoles of chlorogenic acid equivalent per gram dry weight.

**Table 3:** The correlationship between total phenolics (TP), total flavonoid (TF), Gluthatione (GSH). Oxidized Gluthatione (GSSH), peroxide radicals (ROO), superoxide radicals (O_2_), hydrogen peroxide (H_2_O_2_) hydroxyl radicals (OH) and sucrose (Suc) in the study.

	1	2	3	4	5	6	7	8	9
**1.TP**	1.00								
**2. TF**	0.97 *	1.00							
**3. GSH**	0.89 *	0.78 *	1.00						
**4. GSSH**	0.87 *	0.87 *	0.88	1.00					
**5. ROO**	0.87 *	0.83 *	0.84 *	0.81 *	1.00				
**6. O_2_**	0.78 *	0.83 **	0.86 *	0.89 *	0.85 *	1.00			
**7. H_2_O_2_**	0.78 *	0.75 *	0.86 *	0.90 *	0.86 **	0.81 *	1.00		
**8. OH**	0.76 *	0.90 *	0.89 *	0.87 *	0.83 *	0.92 *	0.79 *	1.00	
**9. Suc**	0.88 *	0.76 *	0.87 *	0.92 *	0.86 *	0.76 *	0.81 *	0.83 *	1.00

* and ** respectively significant at *P* ≤ 0.05 or *P* ≤ 0.01.

### 2.3. Glutathione (GSH), Oxidised Glutathione (GSSG) and Ratio of GSH/GSSG

The GSH, GSGG and GSH/GSSG in *L. pumila* were influenced by the interaction between CO_2_ and plant parts (P ≤ 0.01; Table 4). The GSH, GSSG and GSH/GSGG ratio were found to have similar trends as total phenolics and flavonoids accumulation. For GSH the highest accumulation of GSH (890.22 nmol gluthathione/g dry weight) was found to be in the leaf at 1,200 µmol/mol CO_2_, while the lowest (552.2 nmol glutathione/g dry weight) was observed in the root at 400 µmol/mol CO_2_. In GSSG, leaf-1,200 and root-400 µmol/mol CO_2_ recorded 178.65 and 98.34 nmol oxidised glutathione/g dry weight, respectively. For GSH/GSSG ratio the leaf-1,200 µmol/mol CO_2_ was statistically significantly higher than leaf-800 and −400 µmol/mol CO_2_. However, leaf GSH/GSSG ratio between 400 and 800 µmol/mol CO_2_ was not statistically significant from each other. GSH is a tripeptide composed of cysteine, glutamic acid and glycine and is the most abundant nonprotein thiol in the cells. Its active group is the thiol (–SH) of cysteine. The majority of GSH is maintained in the reduced state. The GSH plays an important role in the stabilization of many enzymes. In addition, as antioxidant scavenger it serves as a substrate for dehydroascorbate (DHAsA) reductase and is also directly reactive with free radical including the hydroxyl radical to prevent the inactivation of enzymes by oxidation of an essential thiol group [41]. GSSG consists of two GSH molecules joined by their –SH group into a disulfide bridge and was found to be present in low quantities compared to GSH [25].

**Table 4:** Gluthathione (GSH), Oxidised Gluthatione (GSHO) and GSH/GSSG ratio in different part of *L. pumila* under different CO_2_ concentrations.

CO_2_ levels (µmol mol^−1^)	Plant parts	GSH (nmol/g dry wt)	GSSG (nmol/g dry wt)	GSH/GSSG
	Leaf	744.21 ± 7.3b	160.23 ± 24.1a	4.64 ± 1.7b
**400**	Stem	624.21 ± 2.1c	120.23 ± 1.7b	4.10 ± 2.5c
	Root	504.23 ± 21.2d	98.34 ± 1.7c	3.93 ± 0.4d
	Leaf	810.21 ± 3.4a	176.34 ± 2.4a	4.89 ± 0.6b
**800**	Stem	644.21 ± 2.7c	132.12 ± 3.6b	4.87 ± 1.7d
	Root	505.72 ± 3.6d	100.23 ± 2.7c	4.94 ± 2.8a
	Leaf	890.22 ± 2.4a	178.65 ± 2.4a	5.85 ± 2.1a
**1200**	Stem	698.22 ± 1.8b	132.14 ± 3.5b	4.08 ± 1.3c
	Root	554.42 ± 21.3d	99.43 ± 12.3c	5.37 ± 1.4a

All analyses are mean ± standard error of mean (SEM), N = 18. Means not sharing a common single letter were significantly different at P ≤ 0.05.

In the present study, we found that CO_2_ enrichment increased GSH, GSSG and GSH/GSSG ratio. The high GSH and GSH/GSSG ratio are necessary for several physiological functions. Those include activation and inactivation of redox-dependent enzyme systems and regeneration of cellular antioxidant ascorbic acid under oxidative conditions [42,43]. Usually, the increase in GSH and the ratio of GSH/GSSG in enhanced CO_2_ levels was associated with increase in antioxidant properties [25]. In the current study it was shown that GSH and GSSG have a strong positive relationship with phenolics, flavonoids, ROO, O_2_, H_2_O_2_ and OH. The result showed that the increase in antioxidative properties of *L. pumila* under elevated CO_2_ might be due to increase in production of total phenolics, flavonoids, GSH and GSSG activity that can increase the antioxidant of this plant under elevated CO_2_ [44,45].

### 2.4. Soluble Sugar

The accumulation and partitioning of soluble sugar were influenced by carbon dioxide enrichment (*P* ≤ 0.05). The accumulation of soluble sugar in different parts of the plant followed a descending order of leaf > root > stem (Table 5). Under ambient conditions, less sucrose was produced in the leaf, stem and root compared to those plants exposed to high CO_2_ concentration. The present result suggested that enrichment of *L. pumila* under high CO_2_ was able to enhance the soluble sugar [37,38,39,40]. In recent study by Ibrahim *et al.* [33] on *L. pumila* it was observed that the increase in production of total phenolics and flavonoids in *L. pumila* was due to increase in total non-structural carbohydrates that up-regulated the production of secondary metabolites. The same observation was found in the present study. Data from the correlation Table 3 have shown that soluble sugar had a significant positive relationship with the secondary metabolites (total phenolics and flavonoids) and antioxidant properties. The result implied that the increase in secondary metabolites and antioxidative potential of plant under elevated CO_2_ might be due to enhancement of production of sucrose. The present finding was in agreement with Guo *et al.* [46] that found an increase in sucrose content corresponding to the enhanced production of ascorbic acid, glucosinolates, sulforaphane, anthocyanins, total phenolics and increased antioxidative activities in broccoli sprouts. The possitive correlation between carbohydrate content and antioxidative properties in plants were also reported by other researchers [47,48]. The curent results indicate that enhancement of *L. pumila* with CO_2_ enrichment can enhance the health promoting effects of this plant due to the increases in total phenolics, flavonoids, GSH and GSGG contents.

**Table 5:** Accumulation and partitioning of total soluble in different plant parts of *L. pumila* Blume. under different CO_2_ levels.

CO_2_ levels (µmol mol^−1^)	Plant parts	TSS (mg g^−1^ sucrose dry weight)
	Leaf	16.10 ± 0.35c
**400**	Stem	14.12 ± 0.87c
	Root	9.34 ± 0.97e
	Leaf	21.10 ± 0.46b
**800**	Stem	16.36 ± 0.44c
	Root	12.32 ± 0.56d
	Leaf	27.96 ± 0.76a
**1200**	Stem	19.45 ± 0.56b
	Root	14.24 ± 0.11c

All analyses are mean ± standard error of mean (SEM), N = 18. Means not sharing a common single letter were significantly different at P ≤ 0.05.

## 3. Experimental

### 3.1. Experimental Location, Plant Materials and Treatments

The experiment was carried out under a growth house complex at Field 2, Faculty of Agriculture Glasshouse Complex, Universiti Putra Malaysia (longitude 101° 44’ N and latitude 2° 58’S, 68 m above sea level) with a mean atmospheric pressure of 1.013 kPa. Three-month old *L. pumila* var alata seedlings were left for a month in a nursery to acclimatize until they were ready for the treatments. CO_2_ enrichment treatment started when the seedlings reached four months of age where plants were exposed to 400, 800 and 1,200 µmol^−1^ mol^−1^ CO_2_. This 2-factorial experiment was arranged in randomized complete block design with CO_2_ levels and plant parts are the factors and replicated three times.

### 3.2. Growth House Microclimate and CO_2_ Enrichment Treatment

The seedlings were raised in specially constructed growth houses receiving 12-h photoperiod and average photosynthetic photon flux density of 300 µmol m^−2^ s^−1^. Day and night temperatures were recorded at 30 ± 1.0 °C and 20 ± 1.5 °C, respectively, and relative humidity at about 70% to 80%. Vapor pressure deficit ranged from 1.01 to 2.52 kPa. Carbon dioxide at 99.8% purity was supplied from a high-pressure CO_2_ cylinder and injected through a pressure regulator into fully sealed 2 m × 3 m growth houses at 2-h daily and applied continuous from 08:00 to 10:00 a.m. [47]. The CO_2_ concentration at different treatments was measured using Air Sense ™ CO_2_ sensors designated to each chamber during CO_2_ exposition period. Plants were watered three to four times a day at 5 min per session to ensure normal growth of plant using drip irrigation with emitter capacity of 2 L h^−1^. The experiment lasted for 15 weeks from the onset of treatment.

### 3.3. Extract Preparation

Leaves, stems and root were freeze-dried to constant weights prior to being used in the extraction process. For antioxidant analysis, the leaves, stems, and roots were powdered and 1 g of the powder was extracted continuously with methanol (50 mL). The solution was then swirled for 1 h at room temperature using an orbital shaker. Extracts were then filtered under suction and stored at −20 °C for further use.

### 3.4. Total Phenolics and Total Flavonoids Quantification

The method of extraction and quantification for total phenolics and flavonoids contents followed after Jaafar *et al. *[50]. An amount of ground tissue sample (0.1 g) was extracted with 80% ethanol (10 mL) on an orbital shaker for 120 min at 50 °C. The mixture was subsequently filtered (Whatman™ No.1), and the filtrate was used for the quantification of total phenolics and total flavonoids. Folin-Ciocalteu reagent (diluted 10-fold) was used to determine the total phenolics content of the leaf samples. Two hundred micro liter of the sample extract was mixed with Follin-Ciocalteau reagent (1.5 mL) and allowed to stand at 22 °C for 5 min before adding NaNO_3_ solution (1.5 mL, 60 g/L). After 2 h at 22 °C, absorbance was measured at 725 nm. The results were expressed as mg/g gallic acid equivalent (mg GAE/g dry sample). For total flavonoids determination, sample (1 mL) was mixed with NaNO_3_ (0.3 mL) in a test tube covered with aluminium foil, and left for 5 min. Then 10% AlCl_3_ (0.3 mL) was added followed by addition of 1 M NaOH (2 mL) and the absorbance was measured at 510 nm using rutin as a standard (mg rutin/g dry sample).

### 3.5. Measurement of Glutathione (GSH) and Oxidized Glutathione (GSSG)

GSH and GSSG were assayed using the method described by Castillo and Greppin [51]. Total glutathione were determined by reacting plant extracts (0.5 mL) with 50 mM KH_2_PO_4_/2.5 mM EDTA buffer (pH 7.5), 0.6 mM DTNB [5,5-dithiobis-2-nitrobenzoic acid] in 100 mM Tris-HCl, pH 8.0, 1 unit of glutathione reductase (GR, from spinach, EC 1.6.4.2) and 0.5 mM NADPH. GSH was quantified from the reaction mixture by mixing plant extract (0.5 mL) with 60 mM KH_2_PO_4_/2.5 mM EDTA buffer (pH 7.5), 0.6 mM DTNB [5,5-dithiobis-2-nitrobenzoic acid] in 200 mM Tris-HCl, pH 8.0. The mixture was incubated at 30 °C for 15 min, and the reaction was followed as the rate of change in absorbance at 412 nm using light spectrophotometer (UV-3101P, Labomed Inc, USA) GSSG was determined after removal of GSH from the plant extract.

### 3.6. Measurement of Oxygen Radical Absorbance Capacity (ORAC) Assay

The ORAC reaction was carried out in 75 mM phosphate buffer (pH 7.4), and the final reaction mixture was 200 μL as described by Davalos *et al.* [52]. Antioxidant (20 μL) and fluorescein (FL) (120 μL; 70 nM, final concentration) solutions were placed in the well of the microplate. The mixture was preincubated for 15 min at 37 °C. 2-Amidinopropane (AAPH) solution (60 μL; 12 mM final concentration) was added rapidly using a multichannel pipet. The microplate was immediately placed in the reader and the fluorescence recorded every minute for 80 min. The microplate was automatically shaken prior each reading. A blank (FL + AAPH) using phosphate buffer instead of the antioxidant solution and eight calibration solutions using Trolox (1−8 μM, final concentration) as antioxidant were also carried out in each assay. All the reaction mixtures were prepared in duplicate, and at least three independent assays were performed for each sample. The ORAC value refers to the net protection area under the quenching in the presence of an antioxidant. The final results (ORAC value) were calculated and expressed using Trolox equivalents per gram dry weight basis.

### 3.7. Measurement of Superoxide Radical (O_2_) Assay

The assay for O_2_ was done using the method of Wang *et al.* [25]. The O_2_ was generated by xanthine/xanthine-oxidase systems. Nitrite formation from hydroxylammonium chloride was determined at 530 nm in the spectrophotometer. The reaction mixture contained 1.0 mL of 65 mM Na-phosphate buffer (pH 7.8), 0.1 mL of 7.5 mM xanthine, 0.1 mL of 10 mM hydroxylammonium chloride, 0.1 mL of fruit extract, and 0.4 mL of double-distilled H_2_O. The reaction was started by addition of 0.3 mL of xanthine oxidase (containing 60 µg of protein). The total reaction volume was 2.0 mL and incubated at 25 °C for 20 min. Then, 0.5 mL was removed from the above reaction mixture, 0.5 mL of 19 mM sulfanilic acid and 0.5 mL of 1.0%-naphthylamine were added, and the mixture was shaken for 5 min. After standing at room temperature for 20 min, the optical density of the mixture was determined at 530 nm against blanks that had been prepared similarly but without plant extract. The final results were expressed as percent inhibition of O_2_ production in the presence of plant extract. The scavenging capacity of α-tocopherol at various concentrations (1 to 25 µg) on superoxide radical (O_2_) was measured and used for determining the O_2_ scavenging capacity of fruit extract. The antioxidant capacity of fruit extract against the O_2_ value was expressed as µmol of α-tocopherol equivalent per gram dry weight.

### 3.8. H_2_O_2_ Assay Measurements

The hydrogen peroxide measurement was based on that described by Petterson *et al.* [53]. Three hundred milligrams of the youngest, fully expanded plant extract was homogenized in a cold mortar with 5 mL 5% trichloroacetic acid (TCA) containing 0.1 g activated charcoal and 0.1% polyvinyl- polypyrrolidone (PVPP). The homogenate was filtered and centrifuged at 18,000 g for 10 min. The supernatant was filtered through a Millipore filter (0.45 mm) and used for the assay. A 200-mL aliquot was brought to 2 mL with 100 mM potassium phosphate buffer (pH 8.4) and 1 mL of a colorimetric reagent was added. This reagent was prepared daily by mixing 1:1 (v/v) 0.6 potassium titanium oxalate and 0.6 mM 4-2 (2-pyridylazo) resorcinol (disodium salt). After incubating the sample solution at 60 °C for 45 min, the absorbance was measured at 508 nm. Blanks were made by replacing leaf extract with 5% TCA. The antioxidant capacity of plant extract against H_2_O_2_ was expressed as µmole of ascorbate equivalent per gram dry weight.

### 3.9. Measurement of Hydroxyl Radical (OH·) Assay

The assay for OH was done using the methodof Wang *et al.* [25] with slight modifications. The OH in aqueous media was generated through the Fenton reaction. The reaction mixture contained 0.24 M K-phosphate buffer (pH 7.4), 1.0 mM salicyclic acid, 0.3 mM FeSO_4_/EDTA (4 mM), 0.8 mM H_2_O_2_, and 100 µL of extracts. The total reaction volume was 5.0 mL and incubated at 25 °C for 90 min. Then, 120 µL of 6 M HCl was added, followed by extraction in 4 mL of chilled ether. Ether was evaporated to dryness in a water bath at 40 °C, and the residue was dissolved in 1 mL of cold double-distilled water to which the following was added: 0.5 mL of 10% (w/v) trichloroacetic acid in 0.5 M HCl, 1 mL of 10% (w/v) sodium tungstate, 1 mL of 0.5% (w/v) NaNO_2_. After standing for 5 min, absorbance at 510 nm was read immediately after adding 2 mL of 0.5 M KOH. Relative scavenging efficiency (% inhibition of hydroxylation) of plant extract was estimated from the difference in absorbance (OD) with and without addition of the plant extract. The scavenging capacity of chlorogenic acid at various concentrations (1 to 10 µg) on hydroxyl radical (OH ) was measured and used for determining the OH scavenging capacity of plant extract. The antioxidant capacity of plant extract against OH value was expressed as µmole of chlorogenic acid equivalent per gram dry weight.

### 3.10. Soluble Carbohydrates (Sucrose)

Soluble carbohydrates were measured spectrophotometrically using the method described by Edward [54]. Samples (0.5 g) were placed in 15 mL conical tubes. Then distilled water (10 mL) was added and the mixture was then vortexed and incubated for 10 min. Anthrone reagent was prepared using anthrone (0.1 g) that was dissolved in 95% sulphuric acid (50 mL). Sucrose was used as a standard stock solution to prepare a standard curve for the quantification of sucrose in the sample. The mixed sample of ground dry sample and distilled water was centrifuged at a speed of 3,400 rpm for 10 min and then filtered to get the supernatant. To an aliquot (4 mL) of the sample was added anthrone reagent (8 mL) and the mixture was placed in a waterbath set at 100 °C for 5 min before the sample was measured at absorbance 620 nm using UV160U spectrophotometer (Shimadzu, Japan). The soluble sugar in the sample was expresses as mg sucrose per gram dry sample.

### 3.11. Statistical Analysis

Data were analyzed using analysis of variance by SAS version 17. Mean separation test between treatments was performed using Duncan multiple range test and standard error of differences between means was calculated with the assumption that data were normally distributed and equally replicated.

## 4. Conclusions

The application of higher than ambient CO_2_ levels seems able to enhance the production of total phenolics, flavonoids, GSH and GSHH of *L. pumila*. The increase in these compounds in the plant extract might be attributed to the enhanced antioxidative properties of *L. pumila* as indicated by the high oxygen radical absorbance activity against ROO, O_2_, H_2_O_2_, and OH in the plant parts. The study also showed the increase in antioxidative properties of *L. pumila* under elevated CO_2_ might be due to increase in the production of sucrose levels that might enhance the phytomedicinal properties of the plants.
